# Multiscale Data Treatment in Additive Manufacturing

**DOI:** 10.3390/ma16083168

**Published:** 2023-04-17

**Authors:** Damian Gogolewski

**Affiliations:** Faculty of Mechatronics and Mechanical Engineering, Kielce University of Technology, Al. Tysiąclecia Państwa Polskiego 7, 25-314 Kielce, Poland; dgogolewski@tu.kielce.pl

**Keywords:** wavelet transformation, multiscale analysis, surface texture, PBF-LB/M, additive manufacturing

## Abstract

The article assesses the impact of data treatment on the possibility of assessing the morphological features of additively manufactured spherical surfaces. Tests were carried out on specimens manufactured by PBF-LB/M additive technology, using titanium-powder-based material (Ti6Al4V). The surface topography was assessed using one of the multiscale methods—wavelet transformation. The tests carried out on a wide spectrum of mother wavelet forms emphasized the occurrence of characteristic morphological features on the surface of the tested specimens. Moreover, the significance of the impact of specific metrology operations, measurement data processing and its parameters on the filtration result were noted. Comprehensive assessment of additively manufactured spherical surfaces with simultaneous analysis of the impact of measurement data processing is a novelty and fills a research gap relating to comprehensive surface diagnostics. The research contributes to the development of modern diagnostic systems allowing for a fast and comprehensive assessment of surface topography, taking into account the various stages of data analysis.

## 1. Introduction

The dynamic development of science and technology determines the need for significantly more accurate manufacturing of components, but also the development of new algorithms which may be used for their assessment. Component surfaces perform an important function in their perception, which significantly influences their further use, as they actively participate in the process of friction, wear, lubrication [1,2], adhesion or sealing [3]. The morphological features of surfaces strongly influence the possibility to use specific surfaces under real working conditions. An important issue, in this case, is the assessment of the size and the character of specific features. Surface topography is a composite of periodic and non-periodic irregularities, and thus the perception of the surface changes along with the scale of its observation. Specific features become visible at a specific size of scale [4,5]. The multiscale methods developed in recent years allow assessment of a surface’s texture taking into consideration the multitude of specific irregularities. They allow for a more detailed, comprehensive assessment [6,7,8,9]. Over the past years, a number of algorithms have been developed which allow the analysis of surface topography at many scales. The multiscale methods include: geometric methods [10], structural function [11] and sliding bandpass filters [12]. Wavelet transformation is also one of such methods. In this research, wavelet transformation was chosen because it allows analysis of surface irregularity with respect to its frequency, in a specific frequency band. This allowed comparison of discriminate information in the respective bands in order to verify the influence of the impact of particular metrological operations (shape removal, wavelet transformation parameters) on the variation of irregularity distribution. Analysis of the current state-of-the-art shows that it is widely used in a variety of fields [13,14,15,16], including surface metrology [17,18,19,20,21]. It is used for the assessment of surfaces manufactured using conventional methods [22,23] as well as by modern technologies [24,25]. One of the transformation methods includes a discrete approach. The methodology of discrete wavelet transformation is based on applying high- and low-pass filters with specific characteristics allowing assessment of a specific signal in an appropriate frequency band and scale [26,27,28]. The key issue in this particular case is the assessment of the properties of the specific wavelets taking into consideration their potential impact on the obtained filtration results [29,30]. An analysis of the current state-of-the-art has shown that specific properties have a significant impact on the results of the wavelet analysis. The paper [29] evaluates the influence of particular mother wavelets on the results of the edge effect obtained during one-dimensional discrete wavelet analysis. The research has shown that the size of the edge effect created is not equal to the support width of a particular mother wavelet. An analysis of the effect of support width on filtering results was presented in the paper [31]. The research showed that the use of wavelets with a smaller support width results in better, more complex filtering in the initial stages of decomposition which consist of additional high-frequency information. The analysis of the properties of individual mother wavelet forms showed that although the wavelet approach is a lossless transformation (at any level of decomposition we can reconstruct the signal obtaining the input signal), on the basis of the results presented in the paper [30], it can be concluded that for a group of wavelets for which the characteristics of the filters are not exactly similar a different result was obtained. The analysis of the influence of the different mother wavelets is, therefore, an important area of research, and the results presented in the paper provide a better understanding of aspects of wavelet analysis. The use of multiscale methods in surface analysis allows for an increase in the possibility of assessing it [32,33], but also diagnosing the process in a more direct manner [34,35].

The dynamic development of additive technology determines the possibility to use it in an increasingly wide spectrum of applications [36,37]. The ability to manufacture personalised components quickly and accurately, with specific properties or dimensions that are dedicated to a specific user, brings forth a symbiosis of modern manufacturing methods and contemporary industry. The development of additive techniques, new methods and materials used, also based on powdered metals; titanium, among others, ideally fits into this reality. Titanium alloys are used in the manufacture of components such as heat exchangers, in process equipment in the chemical, cellulose, medical, aviation, and automotive industries and in mechanical engineering. A key issue is ensuring the appropriate quality of the specific surfaces, including ensuring the CAD model is mapped over at a high quality [38]. One of the types of surfaces which play a significant role in modern industry includes spherical surfaces. The possibility of additive shaping and the defined positioning of specific dimples and bumps, or the assessment of the components of joints or solids of revolution, are particularly important issues. Additive technologies can be adapted to manufacture a wide array of component types for use in the automotive industry [39,40,41,42], medical industry [43,44], foundry industry [45,46] or pneumatic and hydraulic industries [47]. The key issue here is to take into account the technological parameters of the manufacturing process, as they directly translate to the quality of the components obtained [48,49,50].

It should be noted, based on the current state-of-the-art, that wavelet analysis is an appropriate tool for assessing the surface texture, in particular those manufactured by additive technology, where the classic approach of filtration seems insufficient. It gives potentially great opportunities within the scope of diagnostics and assessment. At present, there is an insufficient number of studies assessing the impact of the specific metrology operations covering measurement data, including wavelet transformation for the purpose of assessing spherical components in the aspect of morphological shape features on the surface, which is a novelty of the work. In addition, the novelty of the work is a comprehensive approach to assessing the impact of the various phases of data treatment and its parameters in aspects of the metrological evaluation of surface topography. Good metrological practice, including the evaluation of the applicability of different types of transformations and algorithms in surface diagnostics, also involves a thorough analysis of the impact of the various stages of data processing on the results of filtration. The research evaluated the impact of the various phases of metrological data treatment on measurement data and their importance in terms of filtration of a surface which contains irregularities and numerous morphological features. The research conducted on a wide spectrum of wavelet transformation parameters highlighted the importance of the influence of individual operations and its parameters on the results of filtering, indicated the differences in individual frequency ranges in the signals resulting from filtering, and identified the occurrence, size and amount of characteristic morphological features on the surfaces of the samples tested. Despite the many research papers related to the analysis of measurement data, there is still a lack of studies that comprehensively evaluate the errors of the filtration process or the information that is filtered out of the profiles in an arbitrary manner despite their potential impact on the subsequent work of components of machine parts. Based on the research, the differences in the distribution of surface irregularities at different phases of analysis were noted, and the influence of the wavelet form on the ability to discriminate between the surfaces, obtained by applying different metrological operations, was determined. A comprehensive evaluation of spherical surfaces additively produced, while analyzing the impact of the processing of measurement data, is a novelty and fills the research gap relating to comprehensive surface diagnostics, and contributes to the understanding of the various stages of evaluation and the filtering of irregularities in accordance with good metrological practice. The research is a contribution to the development of modern diagnostic systems for the rapid and comprehensive evaluation of surface topography, taking into account the particular stages of metrological analysis of measurement data. Tests carried out involving the wavelet transformation enable a comprehensive evaluation of the surface and the development of guidelines for inspecting the process and the surface. The studies fill the research gap in terms of complex analysis of the operation and its parameters within the scope of processing measurement data, performing appropriate filtering, diagnostics and assessment of surfaces.

## 2. Materials and Methods

**Figure 1:**
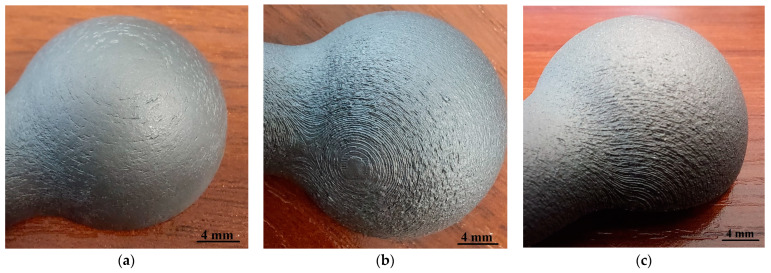
Image of the samples (**a**) 0°, (**b**) 45° and (**c**) 90°.

The specimens selected for testing were designed in SolidWorks 2020 software. The .stl model was then saved using Materialise Magics software. The accuracy was determined in such a way that the .stl file could be processed by 3D printer software. The model was approximated with a linear accuracy combined with an angular accuracy of +/−0.01 mm. The samples were manufactured using PBF-LB/M technology. This technology is based on layer-by-layer model building. The EOS M290 machine software splits the model into layers which are built in an appropriate manner. Responsible for the proper manufacturing process is the G-code, which controls the laser path in order to melt the metal powder and join it with the previously built layer. The .stl file was created with the highest accuracy allowed by the 3D printer software, so the round shape was made with the highest possible accuracy of the 3D printer resulting from technological capabilities (beam diameter, path parameters, grain diameter). The particular layers of powder were melting in the shape of individual model cross sections. The layered nature of the building of the model, and potential process errors, make any nominally round shape formed during the manufacturing process a real polyhedron. This additive technology was selected because of the complex nature of the manufacturing process, and its significant impact on the variability of the surface topography. There are many technological parameters and process phenomena, i.e., powder adhesions, dross formation for downskins, contouring parameters, build geometry, etc., all of which make the mapping of the nominal sphere difficult, and numerous additional features may be present on the surface. An EOS M290 machine was employed to build the samples with specified technological parameters: Inskin laser power—340 W, laser speed—1250 mm/s, hatch distance—0.12 mm, laser spot size—100 µm, layer thickness—60 µm, platform temperature—35 °C, argon was used as a shielding atmosphere and powder fulfills ASTM F1472 and ASTM F2924 standards. To stress-relieve, as instructed by EOS, the manufactured samples were heat treated at 800 °C for 2 h in an argon inert atmosphere. The studies used a titanium powder-based material (Ti6Al4V), produced by EOS. The particle size of the powder was no bigger than 63 µm. The studies used three specimens that had been manufactured at varying angles against the building platform, i.e., 0°, 45° and 90°. This variability was determined by technological limitations and the fact that the printing direction is one of the key parameters affecting the surface irregularity distribution, the occurrence of a staircase effect and the possibility of forming additional features on the surface in the form of, among other things, unmelted powder particles. The images of the samples manufactured at different angles are shown in Figure 1.

**Figure 2:**
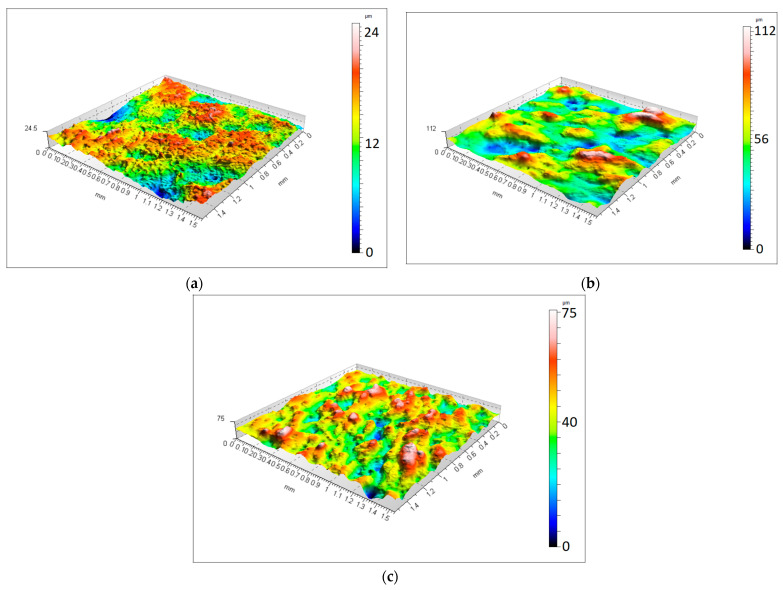
Isometric view of the surface after removal of the shape (**a**) 0°, (**b**) 45° and (**c**) 90°.

The metrological research was performed using a non-contact, optical profilometer Talysurf CCI Lite (Taylor Hobson, Leicester, UK) with a ×10 lens. The area under analysis included surfaces with dimensions of 1.55 mm × 1.55 mm. The surface under assessment was defined as a sphere with a defined radius equal to 14 mm. In order to assess the distribution variability, five measurements were performed for each placement with respect to the building platform. The measurement surface contained a quadrant of the sphere, which was crucial for mapping the last layer during the building process. The multiscale analysis was carried out with the issue of two-dimensional discrete wavelet transformation and a series of wavelet forms: db2, db12, db20, coif2, coif5, sym2, sym8, bior1.5, bior5.5 and dmey. The particular mother wavelets were selected in order to evaluate their properties as widely as possible in terms of their influence on filtering effects. The exemplary isometric views of the measured surfaces after the removal of the shape are presented in Figure 2.

## 3. Results

The research carried out aimed to define the nature, size and amount of the additional morphological features created on the spherical surfaces produced by additive manufacturing as the function of the increment angle and assess of the impact of the transformation applied, depending on the additional operations, on measurement data, i.e., removal of the shape. Wavelet transform tests were carried out on measured surfaces as well as on surfaces where the shape was filtered out. Additionally, for the specific mother wavelets, a statistical analysis using the ANOVA one-way analysis was applied. It was assumed a critical value sufficient to identify similarity was *p* > 0.05.

**Figure 3:**
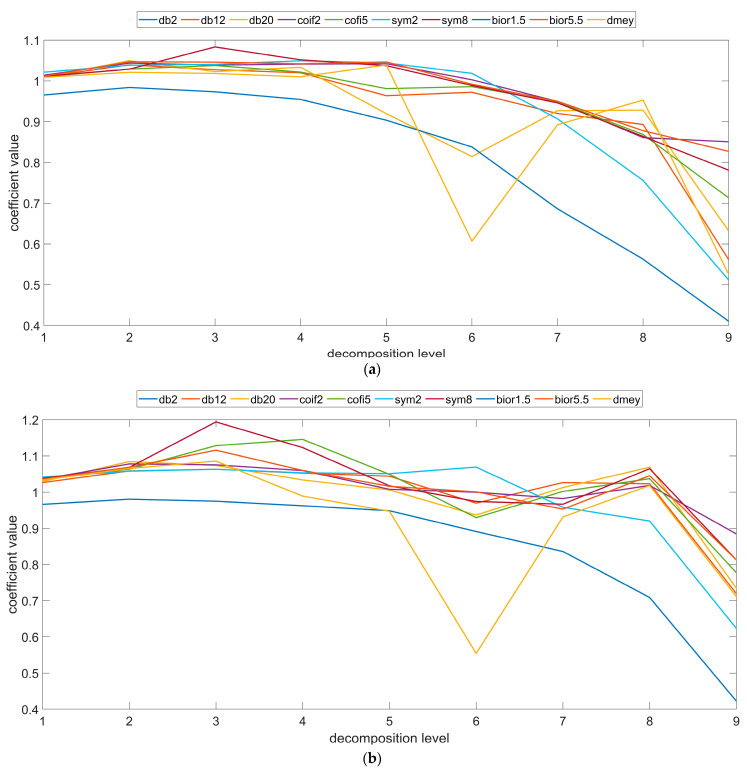

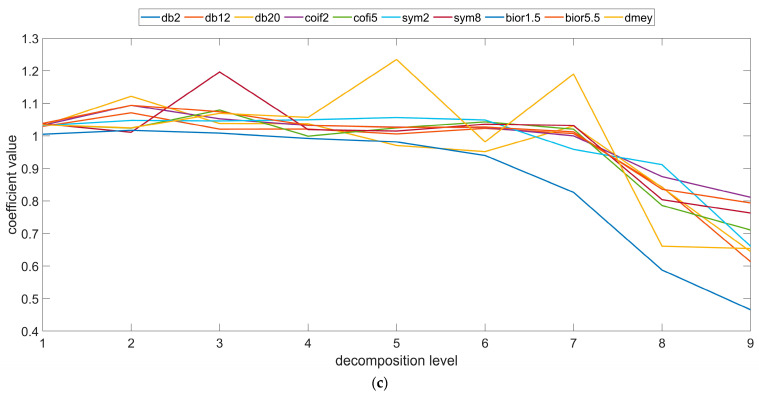
Coefficient value of the quotient calculated for the parameter Root Mean Square Height (**a**) 0°, (**b**) 45°, and (**c**) 90°.

Measurements of the topography of the spherical surfaces revealed the existence of a characteristic distribution of irregularities on the surface. This distribution of irregularities is in line with the direction in which the subsequent layers of materials were applied and related to the technological constraints of the manufacturing process [38,51]. However, the ranges of valleys and peaks which emerged on the surface are not similarly intensively mapped compared to the flat surfaces for the specific building angles [4]. Quantitative and qualitative analyses have shown that the surfaces of the specific specimen also contain additional morphological features resulting from the manufacturing process and powder melting. The size and amount of the specific features depend on the value of the angle at which it is oriented with respect to the building platform. These relationships were noted at each stage of the analysis. At the initial stages for the assessed mother wavelet form, these values are relatively small, only hundredths and thousandths of a micrometer; however, starting from the fifth level of value analysis, these values tend to increase exponentially. A key aspect during such a comprehensive analysis is also determining the impact of the specific metrology operations, performed on the measured data on the final result of the filtration. Figure 3 presents the values of the coefficient determining the quotient of the value of the parameters defining root mean square height determined for the surface for which the shape was removed and the surface measured.

The analysis conducted for the selected forms of mother wavelet showed that at the initial stages of the analysis, the obtained parameter value is close toa value equal to one. This indicated that the filtered high-frequency information for both surfaces (measured and with a removed shape) is similar. For each building angle, the value of the parameter root mean square height is slightly higher for the surface with a removed shape. However, as the decomposition progresses, there is a notable change in the values of the parameter defined by the root mean square height quotient determined for both surfaces. In the remaining details, there is an increasing amount of low-frequency information. The impact of technological constraints, including the layered structure of the sphere, results in obtaining various values of the parameter for each surface. It was noted that all process errors reflected in the surface morphology of the spherical samples are defined and visible at various analysis scale sizes. The differences between the details made during subsequent levels of the analysis highlight the impact of the correct processing of the measurement data. The high-frequency information for both surfaces has been filtered out in an almost analogous manner. Differences for greater wavelengths for individual irregularities were noted.

**Figure 4:**
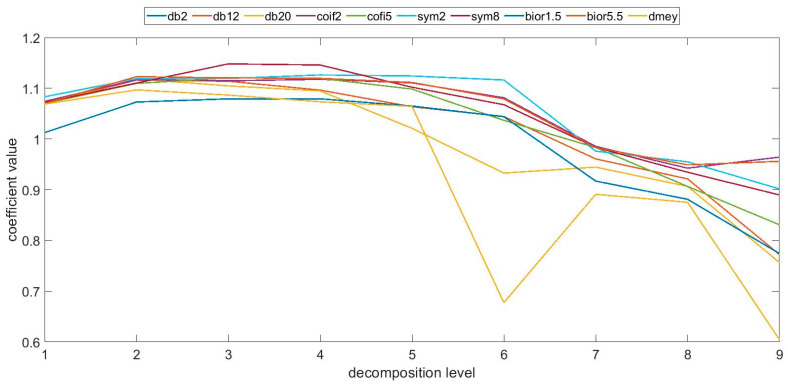
Coefficient values of the quotient calculated for the parameter arithmetical mean height, at 0°.

Tests carried out using other parameters defining surface topography, i.e., arithmetical mean height, skewness and kurtosis showed similar trends for the specific printing directions and analysis levels, in particular for the first one mentioned. The determined coefficient values for the parameters defining skewness and kurtosis show, however, a larger variability in the coefficient value, a greater irregularity and a reduction in the correlation strength. It must be added that the statistical evaluation shows that the variability in the values for all measurements for each sample was relatively small. Exemplary values of the coefficient determined for the arithmetical mean high parameter for the sample made at 0° are shown in Figure 4.

**Figure 5:**
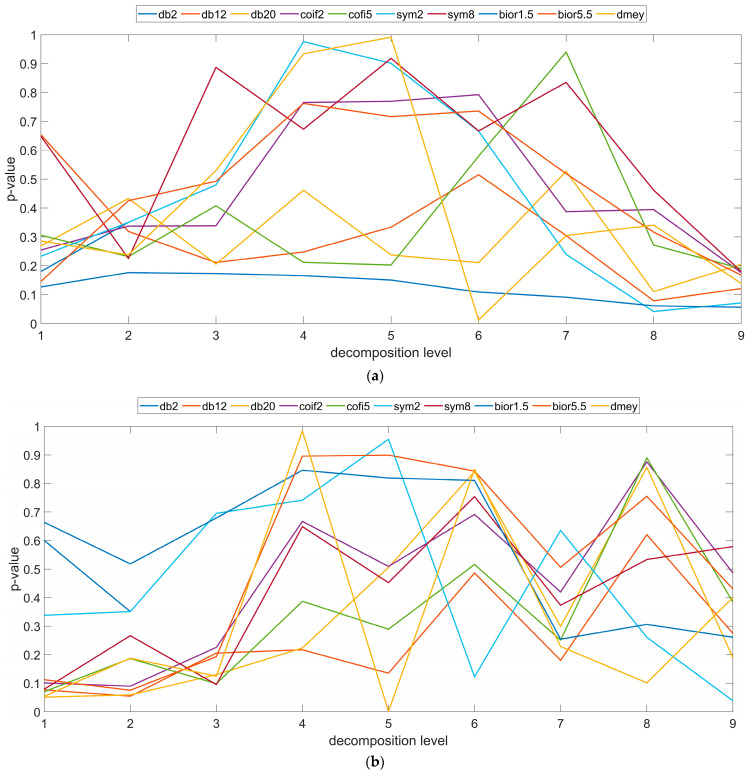

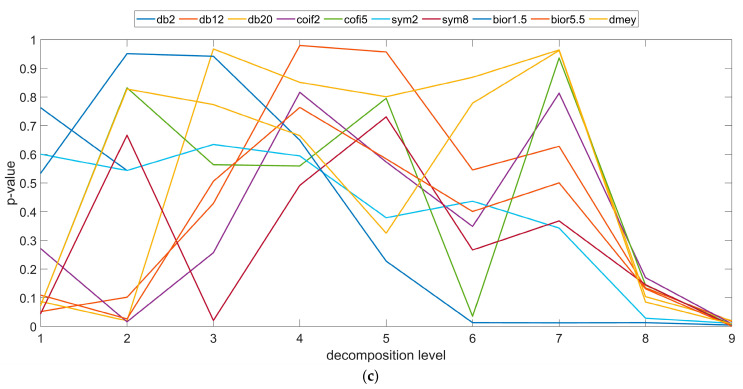
*p*-value coefficient as a function of angle increment to the building platform, (**a**) 0°, (**b**) 45° and (**c**) 90°.

The studies carried out also aimed to assess the impact of the mother wavelet on the potential to filtrate the morphological features of the surface. Based on the determined root mean square height, arithmetical mean height, skewness and kurtosis parameters using the one-way ANOVA (*p* > 0.05) analysis, an assessment was made on the possibility of discrimination of the information obtained on the detail signals on the subsequent stages of analysis. The results of the analysis for the root mean square height parameter are shown in Figure 5. The obtained values emphasize the impact of the wavelet transformation on the obtained results. The study showed a characteristic tendency towards changing the *p*-value parameter for all angles, however, at varying intensities. Despite the fact that there are instances where it is possible to differentiate between the high-frequency signals for the measured surface, with the removed shape for the specific mother wavelets, no significant trend in the aspect of the properties of the specific wavelets has been noted. The lack of similarity visible in the beginning and the final stage of decomposition is random in nature. Regardless, a tendency has been noted that can be defined as a polynomial (parabola) pointing towards the character of alterations at the particular levels of analysis. For individual wavelets it has been noted that during the initial stages of the analysis the *p*-value tends to increase, while reaching lower values at later stages of decomposition (Figure 5). It is particularly apparent for the samples made at an angle of 0° and 90°. This trend is indicative of the fact that different information from both surface topographies was filtered out at the particular levels of analysis. Additive manufacturing errors, such as layered nature, adhesion of unmelted material particles and other defects related to the building process, make it possible for different key aspects of surface irregularities to be noted at different stages of data treatment. For the high-frequency information obtained at the initial levels of analysis, variability in the distribution of irregularities was noted for both 3D distributions. Similarly, lower *p*-values were also obtained at further levels of decomposition. Filtering out higher-frequency information at initial levels, smoothing the surface, allows process defects to be highlighted at these levels that may have been lost during shape removal. This trend is identical to the values shown, among others, in Figure 3. The coefficient value assumes analogous characteristics.

## 4. Discussion

Data treatment is one of the key stages of metrology analysis of measurement data. The development of science and technology determined the need to develop new algorithms which allow for a more complex and comprehensive assessment of a surface, in particular those made by modern methods. Additive technologies, due to technological limitations, and in spite of the numerous advantages [36,52], are not free from disadvantages in particular in terms of surface morphology [53]. In this case, the application of a classic approach [54] often appears insufficient [55]. In recent years, the newly developed solutions, algorithms used in surface metrology in data processing, multiscale [5] or hybrid methods [31], make for an effective alternative that is increasingly more often used in 2D and 3D surface analysis. The new aspect of the research is the comprehensive assessment of the spherical surfaces manufactured additively with a simultaneous analysis of the influence of additional operations and measurement data processing. The studies fill a research gap in terms of comprehensive, in-depth diagnostics and surface evaluation.

Understanding of the impact of data treatment, the impact of its parameters and the possibility of using modern, multiscale methods of measurement data assessment, in respect to the analysis of morphological data, the evaluation of errors and failures of the additive manufacturing process is, without a doubt, a key aspect. This allows for the identification, and the ability of a detailed, in-depth analysis, of the crucial aspects of topography which may have a potential influence on the further use of the specific components. The impact of individual parameters of wavelet transformation in the aspect of identifying these features seems crucial and requires further, wide analysis. The research contributes to the development of modern diagnostic systems allowing for a fast and comprehensive assessment of surface topography, taking into account the various stages of data analysis.

## 5. Conclusions

The paper assesses the impact of data treatment on the possibility of assessing the morphological features of additively manufactured spherical surfaces. The studies took into account the impact of the specific stages of data processing while taking into account the basic operations carried out on the measured measurement data, as well as the use of a modern, multiscale method—wavelet transformation. The studies carried out on a wide spectrum of the mother wavelet emphasized the significance of the impact of individual operations and their parameters on filtration results and identified the occurrence, size and quantity of the characteristic morphological features on the surface of the tested samples. It was noted that at the initial stages, for the selected mother wavelet forms, the filtered high-frequency information indicates that the irregularities are relatively small, hundredths and thousandths of a micrometer; however, starting from the fifth level of value analysis, these values tend to increase exponentially. Quantitative analyses carried out using common parameters used in the evaluation of surface irregularity distributions, i.e., root mean square height, arithmetical mean height, skewness and kurtosis, made it possible to note differences in the distribution of surface irregularities at specific stages of analysis. The variability of these parameters made it possible to assess the nature of irregularities in each scale and building angle. The amount and size of additional morphological features were determined by the value of the building angle, and while analyzing the values of the parameters it should be noted that with the increase of the angle the amount of additional features on the surface increases. Moreover, the impact of the form of the mother wavelet on the possibility to discriminate the surfaces obtained in the results of various, specific metrology operations was defined. The research showed that for the evaluated wavelet forms there was no significant effect of the type of wavelet or its properties. Only sporadic cases of the possibility of discrimination of high-frequency information for the analyzed surfaces were noted. Comprehensive assessment of additively manufactured spherical surfaces, with simultaneous analysis of the impact of measurement data processing, is a novelty, and fills a research gap relating to comprehensive surface diagnostics. The research contributes to the development of modern diagnostic systems allowing for a fast and comprehensive assessment of surface topography, taking into account the various stages of data analysis.

## Data Availability

The data presented in this study are available upon request from the corresponding author.
